# An exploratory cross-cultural study of community-based health literacy interventions to promote the mental well-being of disadvantaged and disabled young Africans: a multi-method approach

**DOI:** 10.3389/fpsyt.2024.1424836

**Published:** 2024-10-23

**Authors:** Darren Sharpe, Mohsen Rajabi, Liliana Galicia Mesa, Ainul Hanafiah, Chinwe Obuaku-Igwe, Julia Davidson, Katongo Chileshe

**Affiliations:** ^1^ Institute for Connected Communities, University of East London, London, United Kingdom; ^2^ Department of Psychology, University of Bath, Bath, United Kingdom; ^3^ Department of Anthropology and Sociology, University of the Western Cape, Cape Town, South Africa; ^4^ Department of Gender Studies, University of Zambia, Lusaka, Zambia

**Keywords:** health literacy, mental health, psychosocial effects, disadvantaged and disabled, community-based interventions, COVID-19, young Africans, LMICs

## Abstract

**Background:**

This study explores the impact of community-based health literacy interventions on the health and mental well-being of disadvantaged young Africans in Zambia, Sierra Leone, Rwanda, and South Africa. The pandemic has exacerbated mental health problems amongst children and young people, underscoring the urgent need for equitable access to mental healthcare resources. Emphasizing the importance of equitable access to mental healthcare resources, the research addresses educational and income disparities in low- and middle-income countries (LMICs), particularly amongst young Africans with disabilities or vulnerabilities.

**Methods:**

The study comprised 1,624 young Africans, of whom 1,592 were surveyed, while 191 later participated in in-depth interviews and focus groups. Additionally, 32 participants exclusively joined focus groups. Participants were recruited to complete the Short Warwick-Edinburgh Mental Well-being Scale (SWEMWBS), WHO-5 Well-being Index, Pandemic Anxiety Scale (PAS), and Self-rating of Happiness, as well as semi-structured interviews or focus groups.

**Results:**

The findings highlight the significance of tailored health literacy interventions in addressing mental health challenges and promoting well-being in marginalized African communities. In the sample, 43.1% (686) reported to have at least a physical disability or serious health condition and 51.4% (818) had special educational needs (SENs). Mental health scores were significantly lower in participants with two or more physical disabilities and pre-existing mental health problems. Factors significantly associated with poor mental health included poor health literacy, physical disabilities, and pre-existing mental health problems. Four main themes were generated from the thematic analysis: early childhood trajectories and mental illness experiences; positionality, open communication, and mental illness experience; mental illness experience, emotional honesty, and social stratification; and spirituality, cultural beliefs, and mental illness experience.

**Conclusion:**

The study emphasizes the need for context-specific, culturally appropriate health literacy interventions to support the mental health and well-being of young Africans in LMICs. By focusing on the lived experiences of disadvantaged groups, the research contributes to a better understanding of effective strategies for promoting health literacy and addressing health inequalities in African communities during and after health emergencies.

## 1 Introduction

This study looks at how community-based health literacy projects, created with young Africans, have affected their health and mental well-being during and after the COVID-19 pandemic. Hereafter, for simplicity, the paper uses the acronym ‘CAYA’ to describe young Africans who are disadvantaged children, adolescents and young adults aged between 12 and 25. The findings stress the need for fair access to mental healthcare resources, especially for CAYA living with disabilities and disadvantaged, while also tackling gaps in education and income. The study points out areas where mental health support needs improvement, noting that this is not always linked to socio-economic differences in low- and middle-income countries (LMICs).

The COVID-19 pandemic has caused a range of problems for CAYA, including changes in their education, social interactions, emotions, and mental health (1–3). They have had to cope with being isolated from others, and with unexpected changes in their daily routines (4, 5). Even though they are less likely to get seriously ill from COVID-19, children and young people have still faced challenges, such as increased domestic violence, stress, and anxiety (1, 6). Prior to the COVID-19 pandemic, young people in Africa were already socially, economically, and politically disempowered, facing significant hardships and stress, particularly regarding education and their transition into meaningful work (7). COVID-19 restrictions further worsened the health and well-being of young Africans, especially those living with disabilities (4), residing in remote rural areas (8), and those constrained by patriarchal forces and deep-seated traditional views that see children as subservient (9). As a result, the mental health of these young individuals deteriorated, with three out of five young Africans expressing a desire to leave the continent, according to the African Union (10, 11). This is evident in LMICs, where existing stressors made the pandemic restrictions even harder for CAYA and their families (12).

This qualitative and quantitative study highlights the vulnerability of specific groups within the population, notably CAYA living with disabilities and/or disadvantaged conditions, who have encountered considerable challenges during the pandemic. These include uncertainty about returning to school or university, and a lack of clinical guidance on staying safe from the virus and coping with pandemic-related restrictions (4). Despite ongoing concerns about their mental health and well-being needs, few studies have addressed the specific needs of this disadvantaged group during and after the pandemic (13–16). Drawing on tried and tested community-based approaches co-developed with Sports in Action, Practical Tools Initiative, Haguruka, Indima Yethu, University of the Western Cape and Njala University, the study combines a range of participatory approaches to better understand the health and welfare needs and priorities of young Africans. These include efforts to enhance their physical and mental health, ensure their overall well-being, and safeguard their digital safety. Additionally, the goal of the project was to facilitate connections between grassroots organisations, enabling them to pool resources and benefit from best practice to better support and protect the health and well-being of CAYA during this unprecedented health emergency.

The current study explores how these initiatives have enabled and empowered CAYA to voice their health and well-being concerns. By focusing on the lived experiences and subjective perceptions of CAYA living with disabilities and/or disadvantaged conditions, this study offers insights into the effectiveness of health literacy interventions in addressing mental health challenges and promoting well-being in marginalized African communities. Prior to the COVID-19 pandemic, our pre-existing research partnership spanned the countries mentioned above, involving local universities – including the University of East London (UEL) in the UK – and local charities. This partnership aimed to test and trial community-based interventions for children and young people in disadvantaged and marginalized communities. These sites were selected due to their significant challenges, including high levels of poverty, limited access to mental health services, and complex social dynamics that impact the well-being of young people. This collaboration has now evolved into the African Youth Safeguarding Network (AYSN), focusing on safeguarding and improving the mental health of youth across these regions. The findings build understanding of the role of health literacy in mitigating the adverse psychosocial effects of the pandemic on mental health amongst young Africans, particularly those living with disabilities and disadvantaged, and they stress the importance of tailored interventions in supporting their needs. According to the African Union, the impact of multidimensional poverty significantly hinders the ability of young Africans to build resilience and maintain good mental health. For example, there is a severe shortage of self-help websites to support young people in developing health literacy. Both public institutions and civil society have lagged behind, failing young Africans, as shown by the lack of authoritative health databases that equip researchers and practitioners to respond to the changing and complex mental health needs of young people across the continent.

This study highlights the disproportionate impact of the COVID-19 pandemic on LMICs due to weaker health systems and economies heavily reliant on sectors affected by the pandemic. The conceptualization of ‘health literacy’ as an asset (detailed later) is used to help mitigate the effects of low literacy through modified communications and improved service organisation. Health literacy is commonly defined as possessing the appropriate skills, knowledge, understanding, and confidence to access, understand, evaluate, use, and navigate health and social care information and services, and it is seen as crucial for addressing health inequalities.

The paper describes community-based interventions rapidly implemented during the pandemic, targeting underserved CAYA and their families. These interventions were developed collaboratively with local communities, ensuring cultural appropriateness, accessibility, and purposefulness in addressing COVID-19 misunderstandings. For instance, tested social media platforms and quasi-public forums were utilized to help disseminate relevant public health messages. Through a co-creation process involving local communities and coordinators, interventions were tailored to fit unique community needs. The monitoring of these interventions indicated increased knowledge and awareness of COVID-19 amongst beneficiaries, with positive feedback from communities and coordinators on the culturally appropriate approach.

Our study also highlights the importance of working together with local communities to create public health programmes that work across different cultures. By collaborating with local organisers and communities, these programmes were developed in a way that really connected with the people they were meant for, helping to improve their understanding of COVID-19 and health in general. In this paper, we argue that it is crucial to focus on health literacy, and to make sure that interventions are culturally appropriate to lessen the effects of the pandemic, especially in low- and middle-income countries.

The pandemic has greatly affected people worldwide, including young individuals in Rwanda, South Africa, Zambia, and Sierra Leone. This study used both qualitative and quantitative methods to understand how the pandemic has impacted CAYA in these countries socially, personally, and physically. It also looked at their health literacy strengths and weaknesses to fight the pandemic.

The paper highlights the significant challenges that CAYA have faced due to the pandemic, such as mental health issues, difficulties accessing healthcare, economic hardships, and social inequalities. Community-based interventions and collaborations between organisations have been crucial in addressing these challenges and supporting disadvantaged populations. To address emerging needs, the project implemented various community-based interventions to improve the health and well-being of CAYA in these countries. These interventions included distributing personal protective equipment (PPE), providing food aid, sharing public health messages, supporting income-generating activities, and offering free health screenings for CAYA with disabilities. Additionally, short training sessions were held for healthcare professionals to improve the customer care environment in health facilities.

The interventions focused on improving ‘health literacy’ by enhancing understanding of COVID-19 amongst CAYA and the wider community. This was achieved through sharing age-appropriate and culturally relevant public health messages on social media, in public talks, through animations, loudspeaker announcements, and mainstream media channels. By providing accurate and accessible information about COVID-19, the project aimed to empower CAYA and their families to make informed decisions about their health and well-being. This study emphasises the importance of tackling the various challenges faced by CAYA in low- and middle-income countries during the COVID-19 pandemic. It highlights the value of community-based initiatives and cross-border collaborations in promoting their health and welfare. By addressing aspects such as mental health, access to healthcare services, and economic empowerment, the project aimed to tackle the underlying causes of health inequalities, and to contribute to sustainable improvements in the lives of CAYA in the participating areas.

The World Health Organization (WHO) defines ‘health literacy’ as the cognitive and social skills that determine an individual’s motivation and ability to access, comprehend, and utilize health information for the promotion and maintenance of good health (17). This definition emphasizes that health literacy goes beyond simply understanding health information; it also includes factors such as motivation and competencies related to health.

Health literacy has evolved from a narrow focus on reading and understanding health information to encompassing a broader range of skills and knowledge related to health (18). It is recognized as a personal resource that empowers individuals to make informed health decisions in their daily lives, and it is considered to be a key determinant of health (19, 20). Accordingly, in this paper, health literacy is understood as an asset (21) performed by research participants to obtain, read, understand, and use healthcare information effectively in order to make appropriate health decisions and to follow instructions for treatment.

In most communities, there are a few main places where people get health information, such as hospitals, schools, public safety groups, and places of worship. But not all communities make it equally easy to get the right information. Communities differ in how many places they have for health information, and how good those places are at giving out different types of information. Some communities have lots of places and resources, such as newspapers, radio and television shows, newsletters, and public libraries, which make it easier to get health information.

A community-based approach to health literacy looks at all the places and ways people get and share health information. It looks at how easy it is to find information in different community spots, so that people can easily find and understand health information to make smart choices about their health and well-being.

The study, which began in 2019 and received funding from the UKRI Global Challenges Research Fund (GCRF), initially aimed to promote social mobility and online safety amongst CAYA in Zambia and Sierra Leone. However, the onset of the COVID-19 pandemic in 2020 led to significant challenges. Lockdown measures meant that partners in Zambia and Sierra Leone could not carry out the programme as planned, disrupting activities and posing threats to the survival of CAYA and their families.

During the lockdown, participants and their families faced various difficulties, including unemployment, financial struggles, and increased health risks due to unequal access to healthcare services. In response, partners in Zambia, Sierra Leone, Rwanda, and South Africa adapted their community-based interventions to target CAYA with disabilities and disadvantaged individuals. The main goal was to educate participants about the risk of the pandemic and preventive measures, as well as to gather information on their needs and priorities through surveys and follow-up interviews. By prioritizing health literacy and meaningful community engagement, the study aimed to address both short-term and long-term challenges faced by CAYA and their families during the pandemic. The community-based interventions aimed to empower participants with knowledge and resources to effectively navigate the challenges of the pandemic.

## 2 Materials and methods

### 2.1 Study design

This study design is an enhancement of phase one, which was rapidly co-developed and applied in Sierra Leone and Zambia at the start of the pandemic to measure the mental health and well-being impact of lockdown measures on CAYA (4). In phase two of the study, which took place during the second wave of the pandemic in Zambia, Sierra Leone, Rwanda, and South Africa, research participants aged 12–25 living with disabilities and/or identified as disadvantaged were involved. Each of the research participants was known to each of the in-country research partners, who ran community-based interventions (e.g., training and education, sports, arts, music, and crafts), coordinated by embedded community workers who held the trust of research participants and their families.

The study design combined quantitative and qualitative methods to identify and understand CAYAs’ different ways of being and seeing, and how they appraised their own mental health and well-being, and to help ascertain how health literacy is applied by those with the least resources. The participatory action study design (22) was suitable for our investigation due to its agility and adaptability to the changing COVID restrictions impacting on how the research sites implemented the research plan, and, importantly, being able to unobtrusively integrate the research tools seamlessly into the community-based interventions. Essentially, the study design recognized that CAYA are a heterogeneous group of people and cultures, and that social and cultural practices influenced the impact/outcome of physical distancing. In practice, the research approach consisted of two stages of data collection: stage one, which was quantitative, followed by stage two, which was qualitative.

During the quantitative stage, we collected self-assessments made by CAYA on their knowledge about, attitudes towards, and practices in terms of well-being and social relationships, using a combination of validated scales. In the qualitative stage, we gathered rich personal accounts into what mattered most to research participants on protective and risk factors to keeping themselves safe from harm, told in semi-structured interviews or focus groups (see **Appendix 1** in **Supplementary Material**). All the datasets underwent either descriptive or thematic data analyses before being synthesized to present a comparative picture of the impact of the pandemic on the daily lives and future wants of community-based support services by young Africans. The project did not directly measure health literacy attainment, but how health literacy resources have been used to help participants to self-care and/or tell their stories of hardship. In the next section, we will discuss each stage of the methodology in more detail.

### 2.2 Ethical considerations

Ethical approval was obtained from the University of East London (UEL) Research Ethics Committee (ETH2021-0159), in conjunction with the University of the Western Cape in South Africa, as well as the Educational Board in Rwanda.

### 2.3 Quantitative phase

#### 2.3.1 Study design and sample

We co-designed and conducted a cross-sectional questionnaire with 1,592 respondents, which was applied in three out of the four countries (South Africa, Zambia, and Sierra Leone). The questionnaire was tested in phase one of the study using the validated Short Warwick-Edinburgh Mental Well-being Scale (SWEMWBS), and we further developed the questionnaire to include the World Health Organization Five Well-being Index (WHO-5), the Pandemic Anxiety Scale (PAS), and the Self-rating of Happiness validated scales to increase the comparative data on how CAYA assess mental health and well-being. Therefore, the goal of the questionnaire was twofold. To have CAYA self-assess their mental health and well-being in response to the restrictions placed on their lives during the pandemic, and to use a cross-cultural lens to help see the commonalities and differences in research participants’ experiences of community-based help and support.

#### 2.3.2 Data collection

Each of the research partners had community workers on the ground who were able to screen potential research participants to ensure that they were eligible to benefit from the community-based intervention, were able and willing to complete the questionnaire – sometimes using a proxy when language was a barrier – and, based on a convenience sample, were available for an in-depth qualitative interview.

#### 2.3.3 Measures

Demographic data were collected, including sex, age, geographical region, nationality, education level (primary schooling, secondary schooling, college, university, other), employment status, history of medical conditions (diabetes, asthma, epilepsy, hearing disability, intellectual disability, visual impairment, physical disability, and clinically diagnosed anxiety or depression), household level of income, and types of special educational needs (communicating and interacting, cognition and learning, social, emotional and mental health difficulties, sensory and/or physical needs).

#### 2.3.4 Short Warwick-Edinburgh Mental Well-being Scale (SWEMWBS)

(23, 24). The shortened version of the Warwick-Edinburgh Mental Well-being Scale (SWEMWBS) was used to measure children’s and adolescents’ mental well-being. The SWEMWBS was designed to assess psychological functioning and emotional well-being. The SWEMWBS comprises seven statements beginning with the phrase “I’ve been”, each with five response options that range from 1 (“none of the time”) to 5 (“all of the time”). The total score ranges from 7 to 35, with higher scores indicating greater mental well-being. Categories for SWEMWBS scores have been established, with a score range of 7–19.3 indicating “low”, 20.0–27.0 indicating “medium”, and 28.1–35 indicating “high” mental well-being. The SWEMWBS is a well-recognized measure for positive mental health and psychological well-being, and it has been used in several low- and middle-income countries (25, 26). The SWEMWBS has been used in various cultural contexts, particularly in Africa (4, 27, 28), and it has proven to be a reliable tool for assessing mental well-being with acceptable psychometric properties in different samples, including construct, content, and criterion validity, as well as internal consistency (α = 0.89).

#### 2.3.5 The World Health Organization Five Well-being Index (WHO-5)

(29). The WHO-5 was used to assess the self-rated psychological well-being of respondents. This scale includes five easy to understand Likert-type statements. Each response is scored on a scale from 0 to 5, with 0 representing “never” and 5 representing “always”. The possible responses in between are “some of the time” (1), “less than half of the time” (2), “more than half of the time” (3), and “most of the time” (4). To obtain a raw score, the responses are multiplied by 4. A raw score of 12.5 or less (total score less than 50) indicates poor well-being and suggests further investigation into possible symptoms of depression.

#### 2.3.6 Pandemic Anxiety Scale (PAS)

(30). In this study, a brief self-report scale was employed to assess concerns regarding the disease and its consequences. The scale measures worries related to contracting COVID-19 (e.g., “I’m worried that I will catch COVID-19”), as well as concerns about the impact of the pandemic and restrictions (e.g., “I’m worried about the long-term effects on my job prospects and the economy”). Respondents rated each item on a 5-point Likert scale, ranging from “Strongly disagree” (0) to “Strongly agree” (4). The total score for the scale ranges from 0 to 36.

#### 2.3.7 The Self-rating of Happiness

To assess respondents’ sense of “happiness”, a single-item measure developed by Abdel-Khalek (31) was employed. Respondents were asked, “Do you feel happy in general?” Happiness was rated on an 11-point scale, ranging from 0 (“not at all happy”) to 10 (“extremely happy”).

To summarise, the research team used the SWEMWBS to measure positive mental health aspects such as coping and optimism, while the WHO-5 Index captured emotional well-being, including mood and life satisfaction. The Pandemic Anxiety Scale (PAS) was employed to assess COVID-related anxiety, and the Self-rating of Happiness was used to evaluate personal happiness. Together, these tools provided a comprehensive assessment of young Africans’ mental health, encompassing well-being, anxiety, and happiness.

#### 2.3.8 Statistical analysis

This study utilized a descriptive statistical approach to analyse data collected from various variables, including demographic information. The response rate was calculated for each item and the total number of answers. Mean (SDs) scores for the SWEMWBS, WHO-5 Index, PAS, and Happiness scale were compared between different groups. Effect sizes were estimated using Cramer’s V (chi-squared tests with categorical variables with more than two categories), Cohen’s d (t-tests with continuous variables), and eta squared (ANOVA with continuous variables). Linear regression was used to examine the relationships between the variables, such as demographic characteristics, history of contact, life satisfaction, social relationships, and mental health-related variables measured by the SWEMWBS, WHO-5 Index, PAS, and Happiness scale. The level of statistical significance was set at *P* < 0.05. All statistical analyses were performed in R.

### 2.4 Qualitative phase

#### 2.4.1 Study design

The study aimed to explore the impact of pandemic restrictions on people’s daily lives, particularly their social interactions and well-being. Qualitative methods were used alongside mental health scales. The study used a purposeful sampling strategy that included registered pupils, students, church members, and beneficiaries connected to the partnership who showed low levels of mental health or who had experienced adverse childhood conditions. Recruitment for the in-depth qualitative interviews continued until no new information was emerging, reaching the saturation point. Interviews and focus group discussions collected narratives on mental illness experiences, healthcare management, and social interactions. Data were coded in NVivo, focusing on aspects related to mental illness and lived realities. Two main themes emerged: contextual/demographic factors and social factors, with subthemes. Insights were analysed using evolutionary psychology categories. A third level of analysis involved comparing these with priorities co-produced with collaborators, yielding themes such as childhood experiences, open communication, social status, and spirituality in relation to mental illness. The study took place over seven months, from March to September 2022. However, qualitative data were collected at different times in each country due to COVID-19 restrictions, the type of interviews (in-person, online, or group), and the characteristics of the participants (e.g., able-bodied, those with intellectual disabilities, undergraduates). Interviews lasted 30 to 60 minutes, including breaks, while focus groups took between 1 to 2 hours. These sessions were conducted in various locations, such as homes, universities, orphanages, and schools. Sometimes, research advocates like teachers and carers helped with sign language and interpreting idiosyncratic language styles. These factors affected how long and when interviews were conducted at each of the four sites.

### 2.4.2 Participants

We utilized semi-structured interviews and focus group discussions to gather data from a total of 223 CAYA aged 12 to 25 across four sub-Saharan African countries: South Africa, Zambia, Sierra Leone, and Rwanda. Of the whole sample, 58% were male, 39% were female, and 3% were categorized as other/prefer not to say (see **Table 1**). The research approach aimed to build understanding of the different perspectives and lived experiences across different demographic groups, including various age ranges and genders, to provide a holistic view of the mental well-being challenges faced by CAYA living with disabilities or vulnerabilities.

**Table 1:** Sample characteristics of qualitative study (N=223).

Characteristics	Zambia (n=25)	Sierra Leone (n=100)	South Africa (n=66)	Rwanda (n=32)
**Qualitative method**	Semi-structured interview	Focus group	Semi-structured interview	Focus group
**Age group**	12–25 years	12–25 years	12–25 years	12–18 years
Gender (%)				
**Male**	15 (60)	50 (50)	49 (74)	15 (47)
**Female**	7 (28)	50 (50)	17 (26)	14 (44)
**Prefer not to say**	3 (12)	0 (0)	0 (0)	3 (9)

#### 2.4.3 Data collection

The qualitative interviews and focus groups were digitally recorded, transcribed, and analysed thematically following the principles of Miles and Huberman (1994) (32). This process involved repeatedly reading the transcripts to become familiar with the content, using coding to identify recurring, similar, and contrasting content, and collapsing the codes into central themes. The cross-cultural research framework was developed and implemented in collaboration with local organisations in Zambia, South Africa, Sierra Leone, and Rwanda. These partners contributed valuable local knowledge and outreach systems to effectively test and expand the initiative to disadvantaged young Africans. Activities varied by country: in South Africa, an undergraduate peer support group was transitioned into a digital peer mentoring support system; in Rwanda, focus groups with students explored perceptions of social media safety during lockdown; in Sierra Leone, COVID-19 health literacy sessions were organised for street homeless youth, including those with disabilities or who were former child soldiers; and in Zambia, CAYA living with disabilities and their families collaborated to share health information and conduct physiotherapy sessions alongside public health campaigns. Demographic questionnaires gathered data on participants’ sex, age, health and well-being, social connections, household size, and economic status. Before the fieldwork, all research assistants were trained to implement the interview schedule, with special attention given to working with children and young people with disabilities. The training ensured consistent understanding of key terms (e.g., “happiness”) across different districts and regions. During fieldwork, the assistants –comprising students and volunteers – were supervised by experienced researchers, and the data they collected were subject to random audits. Participants were provided refreshments to ease stress and to encourage open sharing of their COVID-19 experiences. When language barriers arose, significant adults or researchers who spoke the participant’s dialect provided support. Key questions were prioritized for participants with short attention spans. After each interview, debriefing sessions allowed for reflection on the data collected, minimizing potential bias. Non-English recordings were translated by local teams, with the translations integrated into a central database for analysis. Coding and theme generation were conducted collaboratively by the partnership, ensuring a thorough and consistent approach to data analysis.

#### 2.4.4 Data analysis

Before coding interviews in NVivo, each in-country principal investigator deidentified and scanned interviews, generated codes, and developed potential themes. Thematic analysis followed the six-steps framework suggested by Braun and Clarke (33): 1) Familiarization with data by reading and re-reading transcripts; 2) Assigning codes to meaningful segments; 3) Grouping similar codes to create categories; 4) Clustering categories to form overarching themes, reviewed against original codes and dataset; 5) Naming themes; and 6) Selecting quotations exemplifying each theme from original codes.

Finally, using a social constructionist approach, we collaborated with delivery partners from participating sites to analyse coded data by coders. This led to identifying four key priority areas in participants’ accounts: early childhood experiences, communication and mental illness, emotional honesty and social status, and spirituality and cultural beliefs in relation to mental illness. This analysis aims to understand mental illness complexities and priorities amongst disadvantaged children and youth in sub-Saharan Africa. The collaborative approach ensures that diverse perspectives are considered in interpreting findings.

## 3 Results

### 3.1 Quantitative phase

**Figure 1:**
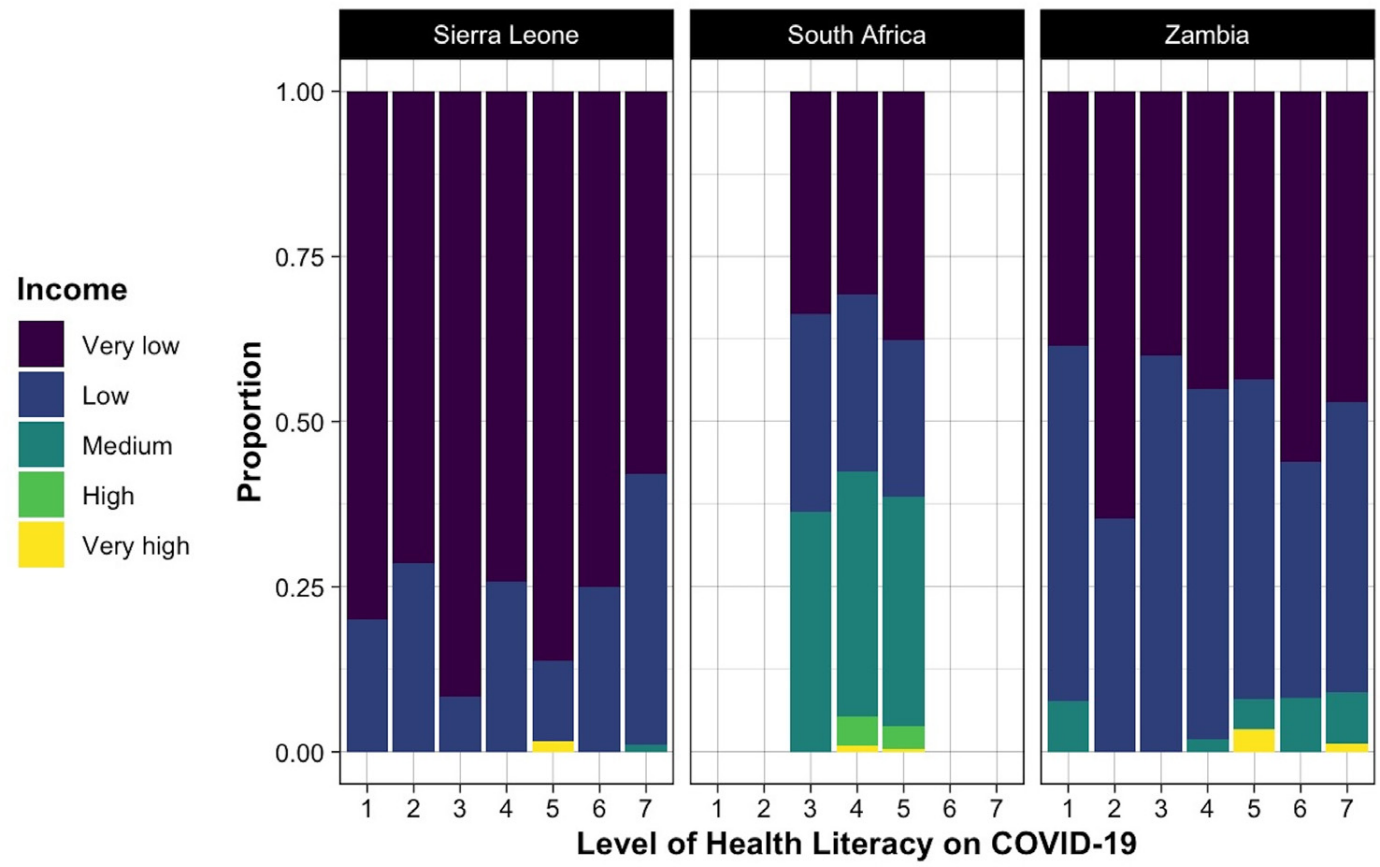
Level of health literacy on COVID-19 across different income levels.

**Figure 2:**
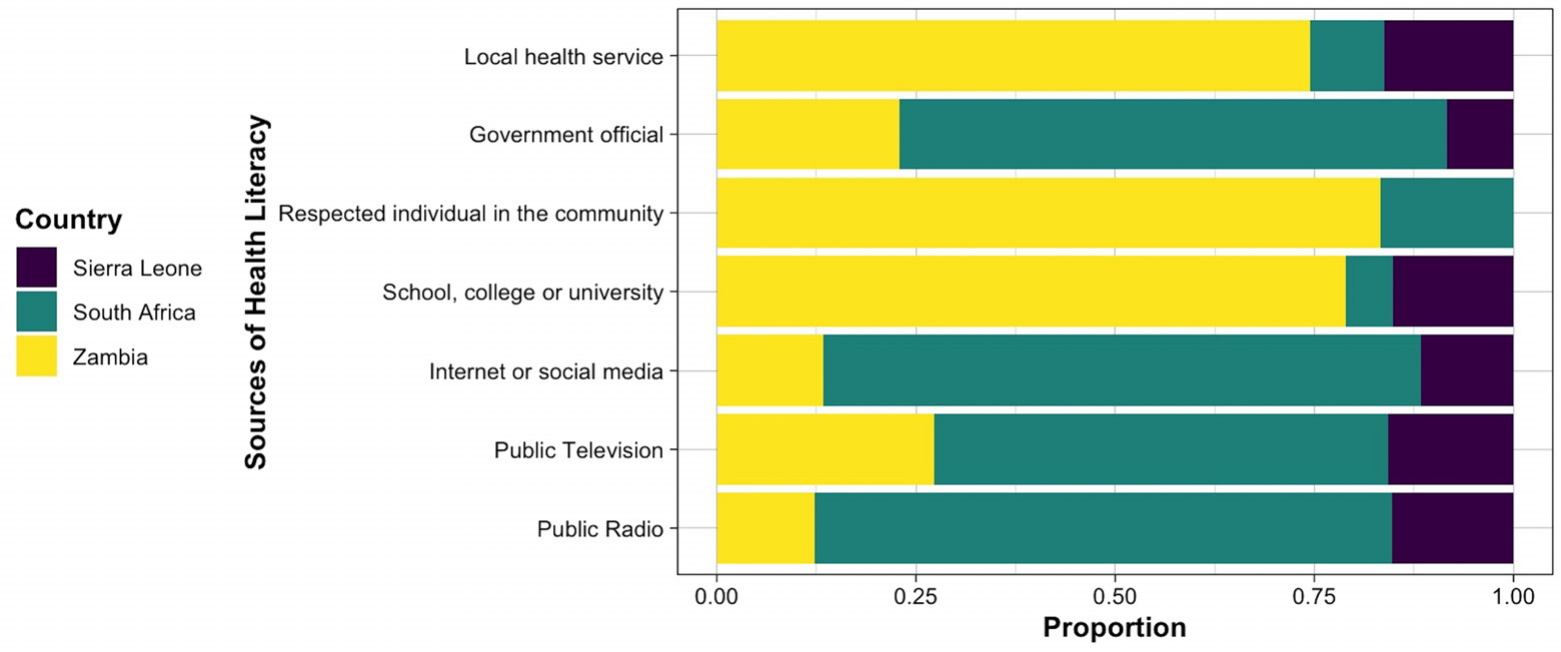
The most common sources of health literacy on COVID-19.

This cross-cultural study included 1,592 participants aged 15 to 25 from Zambia, Sierra Leone, and South Africa. Of the whole sample, about half of the subjects were aged between 21 and 23 years old, and 62.9% (1001) were female. **Table 2** presents demographic information and health conditions of the participants. Amongst the participants, 43.1% (686) reported having at least a physical/intellectual disability or health condition, 51.4% (818) had special educational needs (SENs), and 43.7% (696) classified their income as ‘very low’. The level of health literacy of the participants and the frequency of different sources of health information on COVID-19 are visualized in **Figures 1**, **2**, respectively. Mental health scores were significantly lower in participants with two or more physical disabilities and pre-existing mental health problems. The mean (SD) scores of the total score of all scales are presented in **Table 3**.

**Table 2:** Demographic characteristics of survey participants (N=1592).

Characteristic	Category	Total Frequency (%)	Zambia (%)	Sierra Leone (%)	South Africa (%)
**Gender**	Male	578 (36.3)	169 (38.6)	101 (44.9)	731 (78.7)
	Female	1001 (62.9)	269 (61.4)	124 (55.1)	185 (19.9)
	Non-binary gender identity	9 (0.6)	0 (0)	0 (0)	9 (1)
	Other/prefer not to say	4 (0.3)	0 (0)	0 (0)	4 (0.4)
**Region**	Urban	889 (55.8)	234 (53.4)	175 (77.8)	480 (51.7)
	Semi-urban	455 (28.6)	129 (29.5)	7 (3.1)	319 (34.3)
	Rural	248 (15.6)	75 (17.1)	43 (19.1)	130 (14)
**Age**	15–17 years	470 (29.5)	93 (21.1)	60 (26.6)	317 (34.1)
	18–20 years	252 (15.8)	113 (25.7)	42 (18.6)	97 (10.4)
	21–23 years	763 (47.9)	198 (45.2)	108 (48)	457 (49.1)
	24–25 years	107 (6.7)	34 (7.7)	15 (6.6)	58 (6.2)
**Income**	Very low	696 (43.7)	209 (47.7)	162 (72)	325 (35)
	Low	494 (31)	198 (45.2)	61 (27.2)	235 (25.3)
	Medium	358 (2.5)	26 (5.9)	1 (0.4)	331 (35.6)
	High	33 (2.1)	0 (0)	0 (0)	33 (3.6)
	Very high	11 (0.7)	5 (1.1)	1 (0.4)	5 (0.5)
**Education level**	No qualifications	437 (27.4)	361 (82.4)	76 (33.8)	0 (0)
	At school till aged 16	222 (13.9)	52 (11.9)	24 (10.7)	146 (15.7)
	A-levels (till aged 18)	446 (28)	19 (4.3)	36 (16)	391 (42.1)
	Undergraduate degree	482 (30.3)	6 (1.4)	84 (37.3)	392 (42.2)
	Postgraduate degree	5 (0.3)	0 (0)	5 (2.2)	0 (0)
**Medical condition**	Diabetes	12 (0.7)	7 (1.5)	2 (0.8)	3 (0.3)
	Asthma	93 (5.8)	7 (1.5)	5 (2.2)	81 (8.7)
	Epilepsy	11 (0.6)	7 (1.5)	4 (1.7)	0 (0)
	Hearing disability	37 (2.3)	33 (7.5)	2 (0.8)	2 (0.2)
	Intellectual disability	44 (2.7)	41 (9.3)	1 (0.4)	2 (0.2)
	Visual impairment	123 (7.7)	33 (7.5)	2 (0.8)	88 (9.4)
	Physical disability	92 (5.7)	30 (6.8)	56 (24.8)	6 (0.6)
	Two or more disabilities	50 (3.1)	49 (11.1)	1 (0.4)	0 (0)
	Anxiety/depression	418 (26.2)	41 (9.3)	37 (16.4)	340 (36.5)
	None of the above	906 (56.9)	269 (61.4)	152 (67.5)	485 (52.2)
**Employment**	At university	758 (47.6)	3 (0.7)	82 (36.4)	673 (72.4)
	Self-employed	55 (3.5)	13 (3)	39 (17.3)	3 (0.3)
	Part-time employment	120 (7.5)	13 (3)	4 (1.8)	103 (11.1)
	Full-time employment	52 (3.3)	3 (0.7)	3 (1.3)	46 (5)
	Unable to work	27 (1.7)	26 (5.9)	1 (0.4)	0 (0)
	Homemaker/full-time parent	21 (1.3)	13 (3)	1 (0.4)	7 (0.8)
	Unemployed	559 (35.1)	367 (83.8)	95 (42.2)	97 (10.4)
**SENs**	Yes	818 (51.4)	184 (42)	94 (41.8)	540 (58.1)
	No	774 (46.8)	254 (58)	131 (58.2)	389 (41.9)
**SEN types**	Communicating and interacting	272 (17.1)	33 (18.4)	45 (39.1)	194 (35.7)
	Cognition and learning	105 (6.6)	39 (21.8)	21 (18.3)	45 (8.3)
	Social, emotional, and mental health difficulties	320 (20.1)	20 (11.2)	29 (25.2)	271 (49.9)
	Sensory and/or physical needs	140 (8.8)	87 (48.6)	20 (17.4)	33 (6.1)
**Support**	Yes	1003 (63)	284 (64.8)	130 (57.8)	589 (63.4)
	No	589 (37)	154 (35.2)	95 (42.2)	340 (36.6)
**Support stopped**	Yes	960 (60.3)	289 (66)	127 (56.4)	544 (58.6)
	No	632 (39.7)	149 (34)	98 (43.6)	385(41.4)
**COVID-19 knowledge**	Poor	42 (2.9)	30 (7)	12 (5.3)	0 (0)
	Moderate	1199 (75.6)	158 (37)	112 (50)	929 (100)
	High	339 (21.5)	239 (56)	100 (44.7)	0 (0)
**Contact with friends**	Less than once a day	551 (34.6)	73 (16.7)	28 (12.5)	450 (49.3)
	Once or twice a day	476 (29.9)	179 (40.9)	110 (49.1)	187 (20.5)
	Several times a day	303 (19)	116 (26.5)	32 (14.3)	155 (17)
	Almost all day	244 (15.3)	70 (16)	28 (24.1)	120 (13.2)
**Change in friendship**	No – it is the same	244 (15.3)	47 (10.8)	25 (11.2)	172 (18.5)
	Yes – I interact with them less	1216 (76.4)	373 (85.6)	168 (75)	675 (72.7)
	Yes – I interact with them more	129 (8.1)	16 (3.7)	31 (13.8)	82 (8.8)
**Social isolation and loneliness**	Not at all	507 (31.8)	172 (39.4)	87 (38.8)	248 (26.7)
	Sometimes	963 (60.5)	240 (55)	124 (55.4)	599 (64.5)
	Always	119 (7.5)	24 (5.5)	13 (5.8)	82 (8.8)

**Table 3:** Means (SDs) scores for the SWEMWBS, WHO-5 Index, PAS, and Happiness.

Characteristic	Category	SWEMWBS			WHO-5 Index			PAS			Happiness		
		Mean (SD)	Effect Size	P value	Mean (SD)	Effect Size	P value	Mean (SD)	Effect Size	P value	Mean (SD)	Effect Size	P value
**Country**	Zambia	20.8 (3.4)	0.36	0.000***	51.1 (21.3)	0.25	0.000***	26.3 (4.9)	0.33	0.000***	4.7 (2.5)	0.27	0.000***
	South Africa	19.1 (3.75)			47.2 (20.2)			25.1 (5.4)			3.8 (1.8)		
	Sierra Leone	24.0 (4.7)			54.7 (27.8)			25.7 (6.3)			2.7 (3.3)		
**Region**	Urban	20.8 (4.4)	0.36	0.129	45.4 (22.9)	0.21	0.008**	25.2 (5.5)	0.12	0.040*	4.8 (2.7)	0.09	0.122
	Semi-urban	20.3 (3.5)			45.9 (20.2)			24.9 (5.0)			5.2 (2.0)		
	Rural	20.4 (3.7)			50.4 (23.6)			24.2 (6.2)			5.7 (2.6)		
**Gender**	Female	20.1 (3.9)	0.14	0.000***	43.2 (21.4)	0.17	0.000***	27.2 (5.4)	0.08	0.006**	5.1 (2.3)	0.02	0.157
	Male	21.7 (4.1)			52.2 (22.8)			24.6 (5.7)			4.2 (2.9)		
	Non-binary gender identity	19.0 (1.0)			53.1 (18)			20.6 (1.8)			5.0 (2.4)		
	Other/prefer not to say	19.2 (2.2)			44.0 (13.8)			21.5 (4.1)			6.0 (1.1)		
**Age**	15–17 years	20.9 (4.0)	0.21	0.004**	52.6 (21.7)	0.11	0.000***	25.8 (5.1)	0.32	0.000***	5.8 (2.6)	0.07	0.001**
	18–20 years	19.8 (3.8)			40.8 (20.4)			25.0 (5.0)			5.7 (2.3)		
	21–23 years	20.7 (4.2)			44.2 (21.3)			24.5 (5.8)			5.1 (2.4)		
	24–25 years	21.1 (3.6)			47.2 (21.9)			24.0 (5.8)			5.3 (2.4)		
**Income**	Very low	21.0 (4.3)	0.41	0.000***	47.8 (23.6)	0.11	0.065	25.4 (5.3)	0.16	0.000***	4.5 (2.7)	0.05	0.000***
	Low	20.5 (3.8)			45.8 (21.1)			25.9 (5.5)			5.1 (2.4)		
	Medium	19.9 (3.8)			44.6 (21.5)			23.0 (5.3)			6.0 (2.0)		
	High	20.3 (3.5)			40.8 (15.9)			19.7 (3.0)			5.1 (2.2)		
	Very high	22.4 (5.3)			54.5 (31.5)			25.6 (4.9)			5.3 (3.8)		
**Education**	No qualifications	21.3 (4.1)	0.01	0.001**	52.7 (22.9)	0.03	0.000***	26.4 (4.9)	0.03	0.000***	4.1 (2.8)	0.06	0.000***
	At school till aged 16	20.4 (3.7)			41.9 (20.0)			25.2 (5.7)			5.1 (2.2)		
	A-levels (till aged 18)	20.2 (4.2)			43.3 (22.3)			24.5 (5.4)			5.4 (2.2)		
	Undergraduate degree	20.5 (4.0)			45.5 (21.6)			23.9 (5.6)			5.6 (2.4)		
	Postgraduate degree	21.5 (2.8)			51.2 (32.5)			24.6 (6.0)			1.4 (2.1)		
**Medical condition**	Diabetes	18.3 (2.7)	-0.64	0.002**	37.2 (22.6)	-0.52	0.037*	24.6 (5.8)	-0.057	0.843	4.3 (3.8)	-0.297	0.506
	Asthma	19.6 (4.1)			41.4 (18.6)			24.7 (5.3)			5.6 (2.1)		0.015
	Epilepsy	21.3 (5.1)			44.7 (22.9)			27.1 (1.6)			3.9 (2.5)		0.126
	Hearing disability	20.2 (2.9)			53.7 (18.4)			25.7 (6.2)			4.6 (2.2)		0.323
	Intellectual disability	21.1 (3.1)			55.3 (16.2)			26.3 (4.1)			4.5 (2.4)		0.138
	Visual impairment	19.9 (2.8)			39.5 (17.6)			26.8 (4.3)			5.0 (2.0)		0.798
	Physical disability	21.3 (4.1)			51.3 (14.6)			26.5 (6.2)			3.1 (3.2)		0.000
	Two or more disabilities	17.9 (2.7)			37.0 (19.1)			28.5 (3.0)			3.3 (2.2)		0.000
	Anxiety/depression	18.1 (3.2)			34.1 (16.0)			27.9 (2.7)			4.4 (2.0)		0.001
	None of the above	21.5 (4.1)			51.2 (23.1)			22.5 (5.6)			5.2 (2.6)		0.016
**Employment**	At university	20.2 (3.92)	0.05	0.000***	43.6 (21)	0.02	0.000***	23.8 (5.8)	0.06	0.000***	5.7 (2.2)	0.09	0.000***
	Self-employed	23.4 (4.99)			50.1 (26.9)			26.7 (5.0)			2.4 (3.2)		
	Part-time employment	21.1 (4.3)			48.0 (22.6)			24.0 (4.5)			5.6 (2.1)		
	Full-time employment	20.1 (3.7)			44.5 (20.8)			24.8 (5.6)			5.4 (2.1)		
	Unable to work due to disability	19.8 (3.2)			49.1 (15.4)			28.9 (4.4)			3.8 (2.5)		
	Homemaker/full-time parent	19.8 (4.5)			41.3 (24.2)			26.8 (6.3)			5.0 (3.4)		
	Unemployed	21.0 (4.0)			49.5 (23.5)			26.2 (5.0)			4.4 (2.6)		
**SENs**	Yes	20.2 (4.0)	0.31	0.000***	44.1 (21.5)	0.11	0.000***	25.0 (5.6)	0.01	0.655	5.1 (2.5)	0.01	0.272
	No	21.1 (4.1)			48.7 (23.0)			24.9 (5.4)			5.0 (2.6)		
**SEN types**	Communicating and interacting	20.4 (3.7)	0.41	0.015*	47.1 (21.8)	0.27	0.000***	25.0 (5.6)	0.15	0.000***	5.3 (2.5)	0.04	0.000***
	Cognition and learning	20.6 (3.6)			49.4 (21.5)			24.8 (6.1)			5.2 (2.8)		
	Social, emotional, and mental health difficulties	19.6 (4.2)			40.0 (21.0)			24.1 (5.8)			5.4 (2.2)		
	Sensory and/or physical needs	20.6 (3.9)			45.9 (21.3)			27.3 (3.8)			3.9 (2.8)		
**Support**	Yes	20.6 (3.1)	0.00	0.772	46.9 (22.6)	0.00	0.160	25.2 (5.3)	0.01	0.023*	5.1 (2.4)	0.00	0.233
	No	20.7 (4.3)			45.3 (22.0)			24.4 (5.8)			4.9 (2.6)		
**Support stopped**	Yes	20.6 (4.1)	0.01	0.574	45.1 (22.0)	0.02	0.008	24.9 (5.5)	0.0	0.889	5.0 (2.5)	0.00	0.972
	No	20.7 (4.0)			48.2 (22.8)			25.0 (5.5)			5.0 (2.6)		
**COVID-19 test**	Yes, diagnosed and recovered	18.6 (3.7)	0.03	0.091	40.3 (20.6)	0.02	0.114	26.0 (6.1)	0.01	0.079	6.0 (2.3)	0.02	0.066
	Yes, diagnosed and still ill	19.2 (0.6)			44.0 (1.4)			26.9 (0.2)			6.0 (0.1)		
	Suspected and recovered	19.3 (3.4)			39.2 (18.4)			24.0 (5.4)			5.7 (1.9)		
	Suspected and still ill	19.1 (3.0)			34.6 (26.6)			26.6 (6.4)			4.0 (2.3)		
	No	20.9 (4.1)			47.8 (22.6)			25.0 (5.5)			4.9 (2.6)		
**COVID-19 knowledge**	Poor	20.5 (5.0)	0.03	0.000***	40.4 (23.0)	0.04	0.000***	26.3 (5.7)	0.00	0.026*	2.9 (2.8)	0.04	0.000***
	Moderate	20.3 (3.9)			44.2 (21.9)			24.7 (5.3)			5.3 (2.2)		
	High	21.9 (4.2)			54.6 (22.0)			25.4 (6.1)			4.5 (3.1)		
**COVID-19 source**	Public radio	20.9 (4.0)	0.02	0.000***	44.4 (21.7)	0.02	0.000***	25.5 (5.5)	0.03	0.000***	4.9 (2.6)	0.03	0.000***
	Public television	20.7 (4.0)			48.6 (23.0)			24.7 (5.0)			4.9 (2.4)		
	Internet or social media	19.7 (3.9)			42.1 (21.3)			23.8 (5.9)			5.7 (2.5)		
	School, college, or university	20.9 (4.0)			51.3 (20.3)			26.6 (5.0)			4.3 (2.4)		
	Respected individual in the community	22.1 (4.0)			50.2 (24.4)			24.1 (7.4)			4.8 (2.6)		
	Government official	19.5 (4.7)			47.4 (26.5)			22.5 (5.8)			5.9 (2.2)		
	Local health service	20.8 (4.0)			51.6 (23.8)			26.1 (4.7)			5.2 (2.8)		
**Contact with friends**	Less than once a day	19.1 (3.4)	0.08	0.000***	38.1 (19.1)	0.09	0.000***	25.2 (5.4)	0.03	0.000***	5.3 (2.3)	0.03	0.000***
	Once or twice a day	21.2 (4.2)			48.3 (22.5)			25.7 (5.4)			4.3 (2.6)		
	Several times a day	21.3 (3.7)			49.2 (22.3)			25.0 (5.4)			5.1 (2.1)		
	Almost all day	22.2 (4.4)			58.0 (22.7)			22.5 (5.7)			5.6 (3.0)		
**Change in friendship**	No – it is the same	20.8 (4.1)	0.01	0.000***	48.5 (22.8)	0.02	0.000***	24.3 (5.6)	0.01	0.000***	5.4 (2.6)	0.01	0.000***
	Yes – I interact with them less	20.4 (4.0)			45.0 (22.1)			25.3 (5.4)			4.9 (2.4)		
	Yes – I interact with them more	22.2 (3.9)			55.1 (21.7)			23.3 (6.1)			5.9 (2.8)		
**Social isolation and loneliness**	Not at all	22.1 (4.2)	0.09	0.000***	53.5 (23.5)	0.06	0.000***	24.3 (5.7)	0.01	0.064	5.2 (2.8)	0.00	0.058
	Sometimes	20.2 (3.7)			44.1 (20.5)			25.2 (5.2)			5.0 (2.3)		
	Always	17.6 (3.5)			33.6 (22.1)			25.9 (6.6)			4.7 (2.8)		

*** Significant at 0.001 level, ** Significant at 0.01 level, * Significant at 0.05 level.

The results of the linear regression outcomes are presented in **Table 4**. Factors significantly associated with lower SWEMWBS scores were country of residence (South Africa: β = -4.23; 95% CI -4.8 to -3.7), age (18–20 years: β = -2.26; 95% CI -2.2 to -0.3), history of clinically diagnosed anxiety or depression (β = -3.09; 95% CI -3.6 to -2.6), two or more disabilities (β = -2.53; 95% CI -1.7 to 0.6), having SENs (β = -0.95; 95% CI -1.3 to -0.6), COVID-19 test outcomes (diagnosed and recovered: β = -2.35; 95% CI -3.2 to -1.5), COVID-19 knowledge (poor: β = -2.66; 95% CI -2.7 to -0.1). Frequency of contact with friends (less than once a day: β = -3.08; 95% CI -3.7 to -2.5); and changes in friendship interaction (interact less: β = -1.83; 95% CI -2.6 to -1.1) also showed significant associations with lower mental well-being.

**Table 4:** Associations between demographic factors and SWEMWBS, WHO-5 Index, PAS and Happiness.

Characteristics	Category	SWEMWBS			WHO-5 Index			PAS			Happiness		
		β (95% CI)	R^2^	AR^2^	β (95% CI)	R^2^	AR^2^	β (95% CI)	R^2^	AR^2^	β (95% CI)	R^2^	AR^2^
**Country**	Zambia	-3.15*** (-3.8,-2.5)	0.12	0.11	-3.55* (-7.1,0.0)	0.05	0.04	0.58 (-0.3,1.5)	0.05	0.04	2.05*** (1.7,2.4)	0.17	0.17
	South Africa	-4.23** (-4.8,-3.7)			-12.64*** (-15.8,-9.5)			-1.65** (-2.4,-0.9)			3.10*** (2.8,3.4)		
	Sierra Leone	reference			reference			reference			reference		
**Region**	Urban	0.37 (-0.2,0.9)	0.01	0.00	-4.95 (-8.1,-1.8)	0.01	0.01	1.01 (0.2,1.8)	0.00	0.00	-0.91 (-1.3,-0.6)	0.12	0.02
	Semi-urban	-0.07 (-0.7,0.6)			-4.49 (-8.0,-1.0)			0.77 (-0.1,1.6)			-0.43 (-0.8,0.0)		
	Rural	reference			reference			reference			reference		
**Gender**	Female	2.81 (-1.1,6.8)	0.04	0.04	-0.79 (-2.4,0.8)	0.04	0.04	3.76 (-1.7,9.2)	0.01	0.01	-0.81 (-3.3,1.7)	0.22	0.02
	Male	4.43** (0.5,8.4)			8.21** (-13.4,29.8)			3.10 (-2.3,8.5)			-1.10 (-3.6,1.4)		
	Non-binary gender identity	-0.26 (-5.0,4.5)			-16.89 (-42.8,9.0)			-0.83 (-7.3,5.7)			-1.00 (-4.0,2.0)		
	Other/prefer not to say	reference			reference			reference			reference		
**Age**	15–17 years	-0.19* (-1.0,0.7)	0.01	0.01	5.36* (0.7,10.0)	0.04	0.04	1.85*** (0.7,3.0)	0.07	0.06	-0.83 (-1.4,-0.3)	0.03	0.02
	18–20 years	-2.26** (-2.2,-0.3)			-7.42*** (-11.4,-1.4)			1.04 (-0.2,2.3)			0.35** (-1.2,0.9)		
	21–23 years	-0.39 (-1.2,0.4)			-3.06 (-7.5,1.4)			0.55 (-0.6,1.7)			-0.23 (-0.7,0.3)		
	24–25 years	reference			reference			reference			reference		
**Income**	Very low	-1.39 (-3.8,1.0)	0.01	0.01	-6.73 (-20.1,6.6)	0.02	0.01	-0.16 (-3.4,3.0)	0.06	0.04	-0.81(-2.3,0.7)	0.06	0.05
	Low	-1.91 (-4.3,0.5)			-8.71 (-22.1,4.7)			0.36 (-2.9,3.6)			-0.20 (-1.7,1.3)		
	Medium	-2.59* (-5.0,-0.1)			-9.92 (-23.3,3.5)			-2.56 (-5.8,0.7)			0.64 (-0.9,2.1)		
	High	-2.10 (-4.9,0.7)			-13.70 (-29.0,1.6)			-5.88 (-9.6,-2.2)			-0.18 (-1.9,1.5)		
	Very high	reference			reference			reference			reference		
**Education**	No qualifications	-0.21 (-3.8,3.4)	0.01	0.01	1.50 (-18.0,20.9)	0.03	0.03	1.84 (-3.0,6.7)	0.03	0.03	2.74 (0.6,4.9)	0.06	0.06
	At school till aged 16	-1.14 (-4.8,2.5)			-9.29 (-28.8,10.3)			0.61 (-4.2,5.5)			3.75 (1.6,6.0)		
	A-levels (till aged 18)	-1.34 (-4.9,2.3)			-7.86 (-27.3,11.6)			-0.03 (-4.8,4.8)			4.04 (1.9,6.2)		
	Undergraduate degree	-1.02 (-4.6,2.6)			-5.70 (-25.1,13.7)			-0.65 (-5.5,4.2)			4.23* (2.0,6.4)		
	Postgraduate degree	reference			reference			reference			reference		
**Medical condition**	Diabetes	-1.84 (-4.1,0.4)	0.18	0.17	-7.97 (-14.2,4.3)	0.11	0.10	-1.20 (-4.3,1.9)	0.03	0.02	-0.50 (-1.9,0.9)	0.05	0.05
	Asthma	-0.71* (-1.5,0.1)			-4.06* (-6.6,-2.6)			-0.41 (-1.6,0.7)			0.51* (0.0,1.0)		
	Epilepsy	1.13 (-1.2,3.5)			-3.13 (-17.0,8.8)			0.79 (-2.5,4.1)			-0.17 (-1.7,1.3)		
	Hearing disability	-0.73 (-2.0,0.6)			4.01 (-3.0,11.1)			0.19 (-1.6,2.0)			-0.02 (-0.9,0.8)		
	Intellectual disability	0.10 (-1.1,1.3)			5.53* (-1.0,12.1)			0.78* (-0.9,2.5)			-0.12 (-0.9,0.6)		
	Visual impairment	-0.51 (-1.2,0.2)			-6.67*** (-9.6,-1.7)			2.13** (1.1,3.1)			-0.15 (-0.6,0.3)		
	Physical disability	0.30 (-0.5,1.1)			2.66* (-1.9,7.2)			2.49*** (1.3,2.9)			-3.92*** (-4.1,-1.4)		
	Two or more disabilities	-2.53* (-1.7,0.6)			-6.74** (-0.6,12.9)			3.46*** (1.9,5.0)			-4.16*** (-5.3,-0.8)		
	Anxiety/depression	-3.09*** (-3.6,-2.6)			-14.80*** (-17.3,-12.3)			0.10 (-0.6,0.7)			0.30 (0.9,0.6)		
	None of the above	reference			reference			reference			reference		
**SENs**	Yes	-0.95*** (-1.3,-0.6)	0.01	0.01	-4.59*** (-6.8,-2.4)	0.01	0.01	0.12 (-0.4,0.7)	0.00	0.00	0.14** (-0.1,0.4)	0.02	0.01
	No	reference			reference			reference			reference		
**Support**	Yes	-0.06 (-0.5,0.4)	0.00	0.00	1.63 (-0.6,3.9)	0.00	0.00	0.84 (0.3,1.4)	0.01	0.00	0.16 (-0.1,0.4)	0.00	0.00
	No	reference			reference			reference			reference		
**COVID-19 test**	Yes, diagnosed and recovered	-2.35*** (-3.2,-1.5)	0.09	0.08	-8.49*** (-12.3,-2.7)	0.02	0.02	1.01 (-0.2,2.2)	0.01	0.00	1.07* (0.5,1.6)	0.02	0.02
	Yes, diagnosed and still ill	-1.72 (-9.6,6.2)			-3.82 (-47.4,39.8)			1.99 (-8.9,12.8)			1.05 (-3.9,6.0)		
	Suspected and recovered	-1.63** (-2.3,-1.0)			-4.56* (-5.1,-5.0)			-0.96 (-1.9,-0.1)			0.79 (0.4,1.2)		
	Suspected and still ill	-1.88 (-4.2,0.4)			-13.15 (-15.8,-0.5)			1.65 (-1.5,4.8)			-0.95 (-2.4,0.5)		
	No	reference			reference			reference			reference		
**COVID-19 knowledge**	Poor	-2.66*** (-2.7,-0.1)	0.13	0.12	-14.14*** (-21.2,-7.1)	0.04	0.04	2.87*** (-2.9,3.2)	0.00	0.00	-4.61*** (-3.4,-1.8)	0.04	0.03
	Moderate	-1.61* (-2.1,-1.1)			-10.34** (-13.0,-7.7)			-0.73* (-1.4,-0.1)			0.79* (0.5,1.1)		
	High	reference			reference			reference			reference		
**Health literacy source**	Public radio	0.11 (-1.2,1.4)	0.02	0.01	-7.17 (-14.1,-0.3)	0.02	0.02	-0.67 (-2.4,1.0)	0.03	0.02	-0.22 (-1.0,0.6)	0.03	0.02
	Public television	-0.08 (-1.3,1.2)			-2.93 (-9.9,4.0)			-1.43 (-3.1,0.3)			-0.27 (-1.1,0.5)		
	Internet or social media	-1.07 (-2.4,0.2)			-9.51** (-16.6,-2.4)			-2.35 (-4.1,-0.6)			0.55 (-0.3,1.4)		
	School, college, or university	0.07 (-1.3,1.4)			-0.29 (-7.8,7.2)			0.44 (-1.4,2.3)			-0.84 (-1.7,0.0)		
	Respected individual in the community	1.27 (-0.5,3.1)			-1.41 (-11.3,8.4)			-2.07 (-4.5,0.3)			-0.38 (-1.5,0.7)		
	Government official	-1.31 (-3.0,0.4)			-4.21 (-13.4,4.9)			-3.67* (-5.9,-1.4)			0.71 (-0.3,1.7)		
	Local health service	reference			reference			reference			reference		
**Contact with friends**	Less than once a day	-3.08*** (-3.7,-2.5)	0.13	0.12	-12.89*** (-14.3,-7.7)	0.09	0.09	2.31 (1.5,3.1)	0.03	0.03	-0.25 (0.6,0.1)	0.03	0.03
	Once or twice a day	-0.97* (-1.6,-0.4)			-9.75* (-13.1,-6.4)			2.77 (1.9,3.6)			-1.25 (1.6,-0.9)		
	Several times a day	-0.87 (-1.5,-0.2)			-8.82 (-12.4,-5.2)			2.07 (1.1,3.0)			-0.48 (0.9,-0.1)		
	Almost all day	reference			reference			reference			reference		
**Change in friendship**	No – it is the same	-1.48* (-2.3,-0.6)	0.01	0.01	-6.62* (-11.4,-1.9)	0.02	0.02	1.03 (-0.1,2.2)	0.01	0.01	-0.52 (-1.1,0.0)	0.01	0.01
	Yes – I interact with them less	-1.83*** (-2.6,-1.1)			-10.06*** (-14.1,-6.0)			1.99 (1.0,3.0)			-0.99*** (-1.5,-0.5)		
	Yes – I interact with them more	reference			reference			reference			reference		
**Social isolation and loneliness**	Not at all	4.52*** (3.7,5.3)	0.09	0.09	19.86 ***(15.5,24.2)	0.06	0.06	-1.62 (-2.7,-0.5)	0.01	0.01	0.54 (0.0,1.1)	0.00	0.00
	Sometimes	2.58** (1.8,3.3)			10.48** (6.3,14.6)			-0.70 (-1.8,0.3)			0.29 (-0.2,0.8)		
	Always	reference			reference			reference			reference		

*** Significant at 0.001 level, ** Significant at 0.01 level, * Significant at 0.05 level.

Participants from South Africa (β = -12.64; 95% CI -15.8 to -9.5), aged 18–20 (β = -7.42; 95% CI -11.4 to -1.4), with a history of mental or physical disability (clinically diagnosed anxiety/depression: β = -14.80; 95% CI -17.3 to -12.3; two or more disabilities: β = -6.74; 95% CI -0.6 to 12.9; visual impairments: β = -6.67; 95% CI -9.6 to -1.7), and SENs (β = -4.59; 95% CI -6.8 to -2.4) showed significant relationship with lower scores of the WHO-5 index. Similarly, COVID-19 test outcome (diagnosed and recovered: β = -8.49; 95% CI -12.3 to -2.7), COVID-19 knowledge (poor: β = -14.14; 95% CI -21.2 to -7.1), health literacy source (internet or social media: β = -9.51; 95% CI -16.6 to -2.4), frequency of contact with friends and changes in friendship interaction (less than once a day: -12.89; 95% CI -14.3 to -7.7); interact less: -10.06; 95% CI -14.1 to -6.0) were also significantly correlated with lower subjective psychological well-being.

The results regarding anxiety scores demonstrated that younger participants (15–17: β = 1.85; 95% CI 0.7 to 3.0) and those with physical disabilities (two or more: β = 3.46; 95% CI 1.9 to 5.0; physical disability: β = 2.49; 95% CI 1.3 to 2.9), and poor COVID-19 knowledge (β = 2.87; 95% CI -2.9 to 3.2) gained significantly higher PAS scores. The results regarding the general happiness showed that participants with physical disabilities (two or more: β = -4.16; 95% CI 5.3 to -0.8; physical disability: β = -3.92; 95% CI -4.1,-1.4), poor COVID-19 knowledge (β = -4.61; 95% CI -3.4 to -1.8), and less interaction with friends (β = -0.99; 95% CI -1.5,-0.5) obtained significantly lower scores of happiness.

### 3.2 Qualitative phase

Research participants shared their experiences of trauma and resilience during the pandemic, showing the complexity of accessing social support and having a supportive network. Understanding these themes is crucial for addressing mental health and well-being amongst young Africans. The study offers insights into the emotional and psychological aspects of mental illness experiences and the role of health literacy in managing them. What follows are the four key priorities that frame how research participants recognize risk and potential protective factors.

#### 3.2.1 Theme1: early childhood trajectories and mental illness experiences

Some research participants experienced worsening mental health and learning difficulties due to the pandemic and related restrictions. Additionally, they reported feeling demotivated, lazy, and prone to procrastination, and lacking consistency and support, which hindered their ability to engage in their studies. The pandemic exacerbated pre-existing mental health challenges for these individuals. Research participants said that the pandemic compounded pre-existing mental health challenges:

“I have Bipolar and the anxiety of COVID-19 has aggravated it … anxiety and depression get worse, anger and frustration between family members increase. It has caused me to have more anxiety and also more frequent depressive episodes.” (Female, aged 18, South Africa)

“I do wake up at the middle of the night and fail to go back to sleep … it is different, certain days I would find it a challenge to go to sleep, you would find that I am awake up to somewhere 03.00 hrs a.m., then I sleep.” (Male, aged 17, Zambia)

“My anxiety is about the restrictions and the mechanism the government has put in place, to fight the pandemic, like closing the borders, enforcement of the use of face mask, and introduction of the vaccine.” (Male, aged 19, Sierra Leone)

Research participants come from a diverse range of family structures, including those who live within the traditional (Western) family structure with both parents and siblings; those with single parents (mainly single mothers) and siblings; those who do not live with their parents, but rather with extended relatives (predominantly grandparents); those who live in Fountain of Hope (i.e., a sporting facility for CAYA) and do not see their parents or carers regularly, as in some instances these (parents/extended family/carers) might live in different places; and those who might be forming their own family structures (living with partners). Moreover, some young people experienced family arguments and fights during the COVID-19 social isolation. Therefore, these family structures and, most importantly, the way members interact with each other, made some research participants report a lack of secure attachment to their parents/relatives (sometimes they felt closer to their teachers), as well as increased levels of stress and anxiety. Research participants highlighted the complexities in family structure, dynamics, and interactions:

“My single mom was still completing her degree last year and is doing her post-grad part-time and working this year.” (Female, aged 19, South Africa)

“I don’t have parents, I live with my grandmother.” (Male, aged 21, South Africa)

“I stay with my Aunt and Uncle.” (Male, aged 14, Zambia)

“I am close to my teachers from here.” (Female, aged 14, Zambia)

“Students do not have these conversations with their parents, instead, they talk to their teachers if they need advice.” (Female, aged 15, Rwanda)

#### 3.2.2 Theme2: positionality, open communication, and mental illness experience

In some instances, research participants said that within their homes pre-existing non-mental health difficulties were experienced, and that the social isolation was beneficial to improve relationships amongst family members. However, many other research participants found it very difficult to engage in their studies online, as they were less focused, did not understand content, and had accessibility difficulties, while some just did not like online learning, and others were hugely affected by their home environment, as arguments and fights often happened, which, in turn, affected research participants’ mental health. Additionally, some research participants experienced financial difficulties, as their parents or carers lost their jobs. The examples below illustrate how research participants described how the challenges in their home environment had been detrimental to their study and mental health:

“It was difficult to engage with school content because everything was moving fast and the deadlines gave me pressure.” (Female, aged 19, South Africa)

“I hated studying from home because my parents weren’t that supportive of my mental health…” (Female, aged 21, South Africa)

“…many times, because they would control each and every movement [arguing with parents].” (Female, aged 14, Zambia)

“There has been many fights as we are all forced to be in each other’s spaces, there is no room for one to be strictly by themselves. We have also fallen deep into depression as there has been job losses and deaths in the family.” (Male, aged 17, South Africa)

“It has increased my mental health issues, and being forced to stay inside with my family, we get fed up with one another and it causes arguments.” (Female, aged 18, South Africa)

“I ask my parents about the things going on online to make them think that I care about what they have to say or that they can trust me, and to show them that I am not hiding things, from them.” (Male, aged 14, Rwanda)

As for social interactions, these changed a lot for most research participants. Despite some participants still being able to speak to or see their friends daily (mainly those living in the Fountain of Hope), most research participants said that the way they used to interact with their friends dramatically changed due to the restrictions related to the pandemic. For instance, they did not see their friends face to face, or as regularly as they used to, making them feel disconnected and isolated; young people missed socializing and spending time with their friends. Others did not have much interaction with their family members during the lockdown. Moreover, some young people felt that they were not supported by their teachers or tutors, which made their education engagement more difficult. Research participants commented about how interacting/not interacting outside their home environment had effects on their mental well-being:

“There has been a big change, we used to gather and chat with them, I have a lot of friends but since the coming of COVID-19, I stay away from them to avoid contracting COVID-19.” (Female, aged 18, Zambia)

“…not very much, since the beginning of coronavirus, so usually we just talk on phone, rarely in person.” (Female, aged 14, Zambia)

“I miss socializing, such as meeting people and having conversations with random people…” (Male, aged, 17, South Africa)

“I use social media to meet and catch up with my friends. It’s also easy to talk to schoolmates that you never talk to while at school.” (Female, aged 15, Rwanda)

A very few young people experienced improvements in their relationships with family members, as spending more and quality time together during the lockdown helped them to get closer to each other, which they perceived as a positive aspect of the social isolation:

“Not much, my immediate family is really close and as long as we have each other we are good.” (Female, aged 18, South Africa)

“The majority of my family are homebodies and have not exhibited being negatively affected by the social isolation. The social isolation has brought us within my immediate family closer.” (Male, aged 21, South Africa)

Some participants mentioned serious mental health problems, such as depression, anxiety, stress, or suicidal thoughts, which they or their families experienced due the social isolation and uncertainty within the pandemic context. Moreover, some young people presented sleeping difficulties, either finding it difficult to fall asleep, or waking up in the middle of the night and struggling to go back to sleep. Some also said that they tended to cry or to sleep a lot to avoid thinking, and to cope with stress anxiety triggered by the pandemic. They expressed feelings of unhappiness, lack of satisfaction, loneliness, isolation, uncertainty about their futures, and low energy. Young people felt that they needed support to handle problems and to make decisions. Research participants shared accounts of blockages to open communication about mental health:

“I didn’t know where to start so I procrastinated which gave me more stress, and I had anxiety and slight depression as well…” (Female, aged 25, South Africa)

“We do not have our own space so we aren’t able to introspect that often, this can undoubtedly have a strain on our mental health and make us despondent and on edge, as well as exhaustion as my brother has anger management issues.” (Male, aged 19, South Africa)

Conversely, while some young people were initially struggling to cope with the restrictions, and to concentrate on their studies, and were tending to procrastinate, and finding it difficult to adjust to online learning and to engage in their studies, they eventually were able to engage and study online, while others could handle problems and were able to solve them easily:

“At the beginning, I did not know how to balance school and home life and it took a toll on my mental health, but I eventually got the hang of it. I was able to engage with all my subjects.” (Female, aged 19, South Africa)

“There are groups on social media that help me to study or make conversations to kill boredom.” (Male, aged 15, Rwanda)

Others were able to fully engage in their studies as they got devices from university to be able to study online, and some found the adjustment process easy and quick; others found online learning easier than they initially expected, and some did not feel that it was different from face-to-face learning:

“The university provided me with a laptop and data so that I can engage with work.” (Female, aged 21, South Africa)

“I feel that there is no difference in my schoolwork.” (Male, aged 18, South Africa)

“…it has allowed me to engage with the content at my own pace as lectures were recorded.” (Female, aged 24, South Africa)

#### 3.2.3 Theme3: mental illness experience, emotional honesty, and social stratification [privilege]

Some participants mentioned negative emotions that emerged due to the social isolation resulting from measures to address the COVID-19 pandemic. These included irritation, frustration, anger, irritability, sadness, loneliness, fear, and restriction. Also, a few participants experienced stress and anxiety which were more related to the personal financial consequences of the pandemic. Some others felt more worried about the long-term impact of the pandemic, particularly on the economy/finances and education. Research participants commented on their emotional honesty:

“We feel restricted and cause irritation.” (Male, aged 17, South Africa)

“The pandemic has severely negatively impacted my mental health. It has increased negative emotions such as anxiety and depression. (Female, aged 22, South Africa)

“…it has made it more unbearable for me to stay alive.” (Male, aged 19, South Africa)

Most young people were concerned about their household income, as they or their parents had lost their jobs or employment stability due to the pandemic, which meant that in some instances both parents were unemployed or working fewer hours; therefore, income was reduced, and they faced financial difficulties. Accordingly, young people were concerned about whether there would be enough money in their households to cover food and other necessities. Indeed, some found a job themselves to support their families. Also, some young people and their families received support by getting food or necessities from external organisations or individuals. Also, some students that attended public schools, colleges and universities were on bursaries. However, many felt that either there was no support at all, or that the support was not financially accessible to all, and some expressed lack of academic support from their teachers or lecturers. Research participants commented on SES and support:

“They no longer work due to COVID.” (Male, aged 15, Zambia)

“…My parent lost their job due to COVID, family struggling, therefore I had to get a job…” (Male, aged 17, South Africa)

“The only parent I have has been unemployed since the start of the pandemic.” (Female, aged 19, South Africa)

Some other young people experienced good, appropriate, and accessible support from professionals such as therapists, doctors, nurses, and psychologists, as well as from churches, community leaders, employers, charities, helplines, and universities:

“The university has been quite helpful and generous in providing facilities and incentives to aid in online learning, such as data and VPN.” (Female, aged 22, South Africa)

Regarding healthcare, when young people were asked about their most recent visit to hospital, and whether they felt comfortable, witnessed mistreatment of others, and whether appropriate attention was offered to them, most mentioned some negative experiences, and unhelpful and limited service, as well as poor facilities. Some did not even have access to healthcare services. Some young people suggested that more attention should be paid to mental health services, and that these should be made available in the healthcare system. Research participants remarked on accessing healthcare, resources and education services based on their social position:

“At home it can only be public clinics, and when it comes to fairness I would say no, because there are only two nurses at the clinic and one is soon to retire, it amazes me how the government is not at least providing more healthcare workers in rural areas, but we hope for the best.” (Female, aged 21, South Africa)

“I witnessed a situation where a small child was seriously sick, and the parents were calling for the health worker, and rudely answered the mother to the small child that she needs to be in line because there is coronavirus.” (Female, aged 19, Zambia)

“I am not on medical aid anymore … we don’t have medical aid and we can’t really go to the doctor when we want to.” (Male, aged 17, South Africa)

Conversely, some other participants had good access to healthcare services, and felt that they received a good service, and were happy and comfortable about the support provided to them in the hospital; some even mentioned that the clinic facilities had improved. In most cases, young people mentioned private healthcare:

“I have access to healthcare and support systems, yes.” I do find them fair and useful.” (Female, aged 17, South Africa)

“Most of my family have medical aid so they have access to good health facilities. Some of them have home gyms so they have continued with their exercise regimens throughout the lockdown. This has helped them cope. My daughter and I exercised (not all the time)! in our living space. I don’t think the public health system in the form of clinics and hospitals are adequate or fair.” (Female, aged 24, South Africa)

Some young people struggled with accessing technological resources to engage in their studies; some used only cell phones, and although not all of them specified the reasons, some demonstrated that this is due to limited access to laptops/computers. Therefore, they had difficulties accessing online education due to lack of internet connectivity or devices. However, some received university support by being provided with technological devices with which they could do their studies, and some either borrowed devices from extended family or friends, or adjusted to be able to access the resources needed:

“I was able to join, but I had to travel to family members who had Wi-Fi and who wasn’t always friendly.” (Male, aged 17, South Africa)

“…When mechanism was put in place for student to have online lectures, but was difficult because of internet connection.” (Female, aged 21, Sierra Leone)

“Also with the COVID-19 pandemic most people have put in a lot of effort in learning how to use the internet.” (Male, aged 16, Rwanda)

“It is parents that give us these gadgets, so they kind of supervise how we use them but some things that we do on the phone they never find out.” (Male, aged 14, Rwanda)

#### 3.2.4 Theme4: spirituality, cultural beliefs, and mental illness experience

Some participants rely on spiritual and religious practices, and take God as their main resource for coping with the pandemic. Some only pray, and some, in addition, listen to music, engage in physical activity, watch television series, read, use social media, play video games, talk to friends, or engage in other activities that help them to feel better. Therefore, young people adopt different coping strategies within the COVID-19 context, including making themselves busy with different activities of their own interests, and with praying. Moreover, participants seem to regularly attend a range of different churches; however, the COVID-19 restrictions meant that they were unable to go to church. Others also engaged in housework activities, such as cooking, gardening, or cleaning. Some young people did not receive public or official support; instead, they helped each other, with family members, friends, and the community (some of them being healthcare professionals). Alternatively, they relied on self-help strategies, such as reading, exercise, meditation, and keeping themselves informed:

“For me, prayer is extremely comforting and calming.” (Female, aged 24, South Africa)

“I love to listen to my music. I see music as medication as it helps to ease the pain or maintain good mental health.” (Male, aged 19, South Africa)

“Spiritual help through church counselling and our medical aid would help us.” (Female, aged 21, South Africa)

“I am a basketballer, so I keep myself busy in engaging in sports, and I also focus on my studies.” (Male, aged 16, Zambia)

The health and mental well-being priorities for CAYA are strongly influenced by various factors, including early childhood trajectories, positionality, open communication, emotional honesty, privilege, spiritual beliefs, and cultural beliefs. These factors are shown to significantly impact the research participants’ mental well-being, and to shape their experiences with mental health. Recognizing and understanding these interconnected aspects is crucial in addressing the mental health needs of CAYA effectively. By considering these factors, we can promote culturally sensitive approaches, foster open dialogue, and provide appropriate support systems that respect individuals’ diverse backgrounds and beliefs.

Certainly, tailoring health literacy resources to specific contexts and cultural variations is crucial for effective communication and understanding. Incorporating local dialects and traditions into these resources, such as animations in Zambia in local languages, can greatly enhance accessibility and relevance to the target audience.

Furthermore, it is important for these resources to be rooted in evidence-based guidance from reputable sources and leading bodies in the field of healthcare. This ensures that the information provided is accurate, reliable, and up to date, which is essential for building trust and promoting informed decision making amongst CAYA and marginalized communities. By considering context specificity, cultural variations, and evidence-based guidance, health literacy resources can effectively bridge communication gaps and enable CAYA to make informed choices about their health and well-being.

## 4 Discussion

To recap, 1,592 CAYA between the ages of 12 and 25 years took part in the study, and accessed tailored COVID-19 information to help keep safe during and after the pandemic. Research findings show that participants from South Africa and Sierra Leone had lower mental health scores, contrasting with those from Zambia, who reported higher levels of pandemic-related anxiety. Sex differences were noted, with non-binary individuals scoring lower on mental health scales compared to male or female participants. Moreover, CAYA with pre-existing mental health conditions such as anxiety or depression also scored lower. Some participants expressed limited knowledge, confusion, or stress related to COVID-19.

Research participants from Rwanda, Sierra Leone, South Africa, and Zambia shared their experiences during the COVID-19 pandemic, highlighting various priorities and challenges. In Rwanda, positive experiences included knowledge sharing and group learning, while negative experiences involved inappropriate behaviours and emotional disturbances. Sierra Leonean students faced connectivity issues for education and accessing essential supplies. In South Africa, serious mental health issues arose due to social isolation, with participants relying on family and friends for support. In Zambia, concerns focused on the long-term effects of the pandemic on the economy and education, with participants adopting coping strategies such as avoiding social interactions and praying. These findings underscore the diverse challenges and coping mechanisms across different African regions during the pandemic.

Access to health literacy resources is considered to be a crucial aspect of public health, alongside health protection and disease prevention. In community-based interventions aimed at promoting health and protecting mental well-being during and after the pandemic, health promotion played a significant role. To achieve this, health literacy resources were integrated into all interventions, taking into account contextual differences and available community assets. These interventions needed to be adaptable to overcome challenges, such as electricity shortages in South Africa and accessibility issues in Zambia. Strategies included shifting from online to in-person coaching (e.g., resulting from “loose” lockdown rules), producing tailored animations, recruiting influencers for social media campaigns, organising community health forums, and utilizing megaphones and vans for information dissemination.

Engaging marginalized communities in community health literacy requires cohesive efforts and collective action by public institutions. However, prior to the pandemic, public institutions in participating sites were generally weak in providing an environment conducive to building community health literacy. This study shows that the negative effects of this weakness were mitigated by the efforts of civil society. Delivery organisations collaborated with civil society, despite challenges such as traditional biases and weak public infrastructure, aiming to build health literacy collaboratively with, by, and for individuals and communities.

This study holds significance for various reasons. It emphasizes the importance of building health literacy amongst CAYA living in LMICs to prevent health issues in adulthood. Effective health literacy in childhood acts as a protective factor in adulthood. However, there is limited understanding of which strategies are effective for children and young people, particularly for disabled and disadvantages CAYA. A number of studies, including Reid et al. (34), Jumbe et al. (35), and Ramos et al. (36), have highlighted the limited literature on the effectiveness of building health literacy in communities, particularly amongst young Africans, with even less research focusing specifically on this demographic. Bröder et al. (37) also note the lack of knowledge and academic consensus regarding the abilities and knowledge required by children and young people to make informed health decisions. However, despite these gaps, Abel and McQueen (38) have explored the meaning of health literacy amongst CAYA during the pandemic (39). They found that CAYA demonstrated the ability to engage in physical and psychosocial activities to appropriate standards, interact with others, adapt to necessary changes, and assert reasonable autonomy to achieve complete physical, mental, and social well-being.

In a similar vein, Aluh et al. (40) discovered alarmingly low levels of mental health literacy amongst surveyed Nigerian adolescents, with family and friends being the most recommended source of help. Ouedraogo et al. (41) explored the use of mobile serious games in healthcare education, particularly focusing on their potential to enhance health literacy in rural Africa, suggesting that such games could improve access to health information and promote healthy behaviour modification amongst adolescents and adults. Sodi et al. (42) assessed the implications of mental health literacy for identifying and treating mental health issues amongst school-going adolescents in sub-Saharan Africa. Additionally, Zita (43) investigated the mental health literacy of South African undergraduate Black students, revealing that 39.1% of participants could identify the major symptoms of common mental health disorders related to substance misuse. Despite these built-in structural inequalities, our study found that using “safe” mobile technology to share health information customized for CAYA could help bridge the gap to promote better psychosocial self-care. However, it is crucial for significant adults to back and strengthen these messages, so that positive behaviours are adopted and maintained, even in the face of cultural biases and taboos.

Addressing inequities in healthcare access, quality, and outcomes requires a fundamental shift in creating health literacy resources, utilizing indigenous methods to effectively convey messages that resonate with young Africans’ social realities. These methods include communal conversations, dance and arts, loudspeaker announcements, social media, and digital tools. Despite these efforts, the findings reveal disparities in mental health scores, with lower scores in South Africa and Sierra Leone, and higher anxiety levels in Zambia. The pandemic worsened unemployment rates and increased the risk of violence, especially amongst children. While some participants experienced positive changes in relationships, many faced challenges, such as parental job loss and increased stress, finding solace in spiritual and religious practices.

This paper underscores the importance of building health literacy as a means to enable and empower CAYA in making informed decisions about their health and well-being. By improving their comprehension of health-related information, CAYA can acquire the knowledge and skills necessary to make informed choices, such as understanding preventive measures, recognizing symptoms, accessing appropriate healthcare services, and adopting healthy behaviours. By promoting health literacy, we can contribute to the overall well-being and empowerment of young Africans.

Additionally, this study presents a blueprint aimed at significantly improving the lives of CAYA living with disabilities or disadvantages in LMICs, aligning with United Nations Sustainable Development Goal 2. Through prioritizing mental healthcare and community-based interventions, the study tests a more sustainable approach to addressing recognition, communication, and help-seeking needs that are often overlooked amongst CAYA. The research aims to drive meaningful change within African communities and beyond, fitting into a paradigm shift that emphasizes listening to and understanding African communities’ healthcare needs. By co-producing models of health literacy tailored to young Africans’ surrounding context, traditions, and beliefs, this study seeks to create accessible, understandable, and applicable healthcare resources aligned with their life-worlds.

### 4.1 Limitations

This study, while not directly measuring health literacy attainment, focused on investigating how health literacy is leveraged to bolster resilience and foster the health and well-being of young individuals facing disabilities and vulnerabilities. Led by grassroots partners, the collaborative efforts employed community health literacy techniques to discern the priorities and needs of research participants during and after the pandemic. Addressing a gap in the literature, this study emphasises the pivotal role of health literacy amongst disabled and disadvantaged children and young people in LMICs, while also highlighting the potential for identifying and utilizing community assets.

### 4.2 Implications

The findings of this study underscore the crucial role of providing health literacy information tailored to the context, particularly concerning COVID-19 and online safety, as a means of empowering CAYA to take charge of their health and safety. Additionally, it emphasizes the importance of promoting mental health literacy amongst educators across various educational institutions, aiming to enhance their ability to support and foster resilience amongst CAYA. Furthermore, it suggests that funders and sponsors should incentivize the formation of local partnerships to leverage community assets for scaling proven psychosocial support and counselling interventions targeted at CAYA. Lastly, it calls for future research to delve into the specific needs and priorities of CAYA, encompassing their educational experiences, social contexts, and personal transitions, in order to better allocate resources to the most marginalized CAYA populations.

## 5 Conclusion

This study underscores the importance of collaboration amongst partners in LMICs to enhance health literacy. It highlights the potential of health literacy in addressing disparities, and empowering CAYA to articulate and seek help for their health, social care, and safety concerns. The findings emphasize the effectiveness of tailored health literacy programmes for this demographic and stress the significance of targeted interventions to address healthcare access disparities. While CAYA face significant challenges during their transition to adulthood, support from the global north can make a difference by uplifting individuals and communities.

By co-creating and delivering new health literacy messages in accessible forms, important health information can be effectively appraised, remembered, and applied. This paradigm shift towards partnerships and inclusivity amplifies the voices of CAYA in need, leading to positive change.

In conclusion, this study aimed to understand and address the urgent health challenges faced by young Africans, especially those who are disadvantaged and living with disabilities. It built upon established community-based approaches, respecting the context and acknowledging the sources of knowledge that shape the worldviews of research participants, including intergenerational, historical, and cultural influences.

## Data Availability

The raw data supporting the conclusions of this article will be made available by the authors, without undue reservation.
