# Supplementary material for: An exploratory cross-cultural study of community-based health literacy interventions to promote the mental well-being of disadvantaged and disabled young Africans: a multi-method approach
Source: Front Psychiatry. 2024 Oct 23;15:1424836. doi: 10.3389/fpsyt.2024.1424836 (PMC11537865; doi:10.3389/fpsyt.2024.1424836)
Supplement: Supplementary file 1 [file DataSheet1.pdf]

Appendix 1

Recovering from COVID-19: An alliance to improve the online and offline health and welfare of vulnerable and disadvantaged children and young people living in Rwanda, Zambia, Sierra Leone and South Africa:

Youth Safeguarding Network (YSN)

Children and Young People Assessment Materials

Version 2
03/08/2021

About You and Your Family

1. Where do you live?
Drop down menu

Rwanda
Sierra Leone
South Africa
Zambia

2. Where is your geographical region located?

Urban
Semi-urban
Rural

3. Your gender:

Female
Male
Non-binary gender identity
Other/prefer not to say

4. Your age:

Please specify:

5. What is your highest level of educational attainment?
Drop down menu

No qualifications
Completed GCSE/CSE/O-levels or equivalent (at school till aged 16)
A-levels or equivalent (at school till aged 18)
Undergraduate degree or professional qualification
Postgraduate degree

6. If you are aged 18 and over please specify what is your employment status?
Drop down menu

At University
Self employed
In part-time employment
In full-time employment
Unable to work due to disability
Homemaker/full-time parent
Unemployed and seeking work

7. What is your current/last place of education?

Multiple responses

State school [e.g. I pay no fees]
Independent school [e.g. I pay some fees]
Special provision school [e.g. I use a wheelchair and/or learning disability]
Home educated
College/University
Out of school/university due to the COVID-19 pandemic

8. Do you have any special educational needs?

Yes
No

If yes:
What type of special educational needs?

Communicating and interacting (e.g. communication problems)
Cognition and learning (e.g. understanding problems)
Social, emotional and mental health difficulties (e.g. mental health problem)
Sensory and/or physical needs (e.g. seeing, hearing and moving problem)

9. In the last 12 months, have you received support for any of the following?

- Mental health/emotional/behavioural difficulties
- Housing supports
- Educational support
- Food aids
- COVID-19 health information

Yes
No

If yes:
By whom (please name the organisation):

Has this support been introduced, reduced, or stopped due to COVID-19 outbreak?

Yes
No

10. What is your household level of income?

Very low
Low
Medium
High
Very high

11. Do you have any of the following medical conditions?
Please select all that apply

Diabetes
Asthma
Epilepsy
Hearing disability
Intellectual disability
Visual impairment
Physical disability
Two or more disability
Anxiety/Depression (clinically-diagnosed)
None of the above

12. Have you had COVID-19 (Coronavirus) over the last 12 months?

Yes diagnosed and recovered
Yes diagnosed and still ill
Suspected and recovered
Suspected and still ill
No

13. Has anyone in your household had COVID-19 (Coronavirus) over the last 12 months?

Yes diagnosed and deceased
Yes diagnosed and recovered
Yes diagnosed and still ill
Suspected and recovered
Suspected and still ill
No

14. How would you rate your knowledge to protect yourself from contracting COVID-19?

1. Very poor knowledge	2	3	4	5	6	7. Very good knowledge

15. Where did you mainly get your knowledge on COVID-19 from?

Public Radio
Public Television
Internet or social media
School, college or university
Respected individual in the community
Government official
Local health service

16. Overall, how satisfied are you with your life nowadays?

0 = Not at all satisfied
1
2
3
4
5
6
7
8
9
10 = Completely satisfied

17. Nowadays, do you feel happy in general?

0 = Completely unhappy
1
2
3
4
5
6
7
8
9
10 = Completely happy

18. How often do you hear from or talk to your friends?

Less than once a day
Once or twice a day
Several times a day
Almost all day

19. Has your interaction with peers changed since last 12 months?

No – it is the same
Yes-I interact with them less
Yes- I interact with them more

20. Do you feel that you lack company, peers and friend?

Not at all
Sometimes
Always

21. Do you feel left out?

Not at all
Sometimes
Always

22. Do you feel isolated from others?

Not at all
Sometimes
Always

23. Do you feel lonely?

Not at all
Sometimes
Always

24. Most young people have occasional arguments with their parents/caregivers or family members. How often do you argue with them during the pandemic?

Most days
More than once a week
Less than once a week
Hardly ever
Never

25. Overall, how close would you say you are to your parents/caregivers?

Not very close
Fairly close
Very close
Extremely close

26. Which of these problems have you had with your sleep?
Select all that apply:

Difficulty sleeping
Waking up in the middle of the night
Waking up in the middle of the night and can’t fall back to sleep
Difficulty waking up
Feel unrested on waking up
None of the above

27. Over the past 12 months, about what time do you go to sleep on a weekday night?

Before 9 pm
9 - 9:59 pm
10 - 10:59 pm

11 - midnight
After midnight

28. The following questions will ask about how you are feeling during the COVID-19 pandemic. **Please answer the extent to which you agree with each statement.**

Item	Strongly disagree	Disagree	Neither disagree/agree	Agree	Strongly agree
I believe that COVID-19 is a very serious issue					
I am worried that I will catch COVID-19					
I am worried that my friends and my family will catch COVID-19					
I am worried that I might transmit the infection to someone else					
I am afraid to leave the house right now					
I am worried that we won't have enough food and other essential items during the pandemic					
I am worried about missing school/university/work					
I am worried about the amount of money we have coming in					
I am worried about the long-term impact this pandemic have on their job prospects and the economy					

29. Have you kept a similar routine to how things were before COVID-19 (e.g. bedtime, mealtimes, sleep, and physical activities etc)?

Not at all
A bit
A lot
Completely

30. In the last 12 months, what has been the most negative impact of COVID-19 for you?

Please specify

The Short Warwick-Edinburgh Mental Well-being Scale (SWEMWBS)

19. The following block of questions is about thoughts and feelings. For each of the following statements, **please select the option that best describes your experience over the last two weeks.**

Item	None of the time (1)	Rarely (2)	Some of the time (3)	Often (4)	All of time (5)
I've been feeling optimistic about the future					
I've been feeling useful					
I've been feeling relaxed					
I've been dealing with problems well					
I've been thinking clearly					
I've been feeling close to other people					
I've been able to make up my own mind about things					

WHO Five Well-being Index

Please indicate for each of the five statements which is closet to **how you have been feeling over the last two weeks**. Notice that higher numbers mean better wellbeing.
There are no right or wrong answers. Do not spend too much time on any statement.

Item	All of the time (5)	Most of the time (4)	More than half of the time (3)	Less than half of the time (2)	Some of the time (1)	At no time (0)
I have felt cheerful in good sprits						
I have felt calm and relaxed						
I have felt active and vigorous						
I woke up feeling fresh and rested						
My daily has been filled with things that interest me						

Qualitative Interview: Semistructured Interview Schedule

1. How do you feel about your knowledge level regarding COVID-19 pandemic?
2. What safety measures have you taken for yourself and for your family safety in COVID-19?
3. Do you think COVID-19 will inflict serious damage in your community (social connectedness), if adequate safety measures are not taken?
4. Do you think you can protect yourself against the COVID-19 and its negative impacts?
5. What are your key fears and anxieties related to COVID-19 and its restrictions?
6. What are the mental health consequences of the COVID-19 lockdown and social isolation you and your family?
7. Did you experienced or heard about any sexual abuse, domestic violence during the COVID-19 outbreak?
8. Are you a looked-after child (e.g. adopted by a relative, friend, or volunteer)?
   a. Are you staying/living with your relatives?
9. Can you tell me a bit about your day-to-day family life during the COVID-19 pandemic?
   a. Are you going to school?
   b. What do you do if you are not going to school?
10. Can you tell me a little bit about what your average school day looked like before the COVID-19 pandemic?
    a. Did you do homework?
    b. Favourite subjects and leisure time?
    c. Do parents work how does school life and home-life meet?
11. Can you talk about how the COVID-19 has affected your schoolwork/college?
    a. Did the school/college talk to you about what was happening at the start?
    b. Was your work life affected?
    c. Were there any emotional effects?
    d. Were you able to engage with school subjects and content?
    e. Who in the household manages the home-schooling?
12. Many families have found home-schooling difficult or strange (some have done it, some have not). Can you tell me if you have been able to keep up with school during the pandemic?
    a. Is there anything that has been particularly helpful to you over the last few months in doing home-school work?
13. Is there anything that has been particularly challenging?
    a. How have you had contact with school?

b. Would you have preferred different contact?

c. How about you did you see your teacher in the last while?

d. Have you any ideas on how we could have made this better for children?

14. Do you use computers tablets and/or the Internet for schooling or attending classrooms? tell me what you like to do on these.
15. If this pandemic happened again what do you think schools, colleges or charities could do to help you?
16. Is there anything that we could do online/offline to help with your schooling, routine life and wellbeing in the future?
17. Are you worried about going back to school?
18. What is the biggest thing you miss about going to school? What don't you miss?
19. Can you talk about how the COVID-19 has affected your well-being and health?
20. Can you tell me what you think the future will look like for you and your family?
21. Would you like to tell me anything important about yourself that you have not had a chance to say or has just occurred to you?
22. How was your copping strategy and what lessons you learnt to deal with the the future crisis/pandemics?
23. How do you cope with anxiety and fear related to COVID-19 pandemic?
24. Do you have any suggestions on how government/NGOs could provide support services for coping with challenges related to this crisis situations?
25. Currently, what sort of help or support is accessible to you and your family to cope with the pandemic situation, support their health and wellbeing? Did you find them fair and useful?
26. In your opinion, what are the needs for future preparedness for any outbreak or crisis that prepare community (trainings, ,workshops, public awareness, equipment, etc)?
